# Stress and Self-Efficacy in Parents/Caregivers and Oral Health of Individuals with Down Syndrome During the COVID-19 Pandemic: A Cross-Sectional Study

**DOI:** 10.3390/ijerph21111497

**Published:** 2024-11-11

**Authors:** Julya Ribeiro Campos, Fernando Oliveira Costa, Ana Cristina Borges-Oliveira, Luís Otávio Miranda Cota

**Affiliations:** 1 Department of Dental Clinics, Oral Pathology and Oral Surgery, Periodontology Division, Federal University of Minas Gerais, Belo Horizonte 31270-901, Brazil; julyaribeirocampos@hotmail.com (J.R.C.); focperio@gmail.com (F.O.C.); 2 Department of Social and Preventive Dentistry, Federal University of Minas Gerais, Belo Horizonte 31270-901, Brazil; anacboliveira7@gmail.com

**Keywords:** COVID-19, Down syndrome, oral health, Perceived Stress Scale, self-efficacy

## Abstract

The family of individuals living with Down Syndrome (DS) often demonstrate high levels of stress associated with the demand for care and difficulties experienced in everyday life. The aim of this cross-sectional study was to assess perceived stress by parents/caregivers of individuals with DS and its association with general perceived self-efficacy and dental outcomes, considering the COVID-19 pandemic’s impacts on family’s daily activities and finances. A sample of 257 parents/caregivers answered a questionnaire with socioeconomic, dental, and behavioral variables and the short version of the Perceived Stress Scale and the General Perceived Self-Efficacy Scale. The sample was divided into three groups based on perceived stress levels. Associated variables were evaluated using multinomial logistic regression (level of significance 5%), adjusting for socioeconomic factors. The mean perceived stress score was 17.84 ± 5.75 (0–39). Medium stress (second tertile) was associated with finger/nail biting in individuals with DS (OR = 2.05; 95%CI 1.04–4.03; *p* = 0.038), difficulty in performing oral hygiene (OR = 2.39; 95%CI 1.23–4.65; *p* = 0.011) and medium and high self-efficacy (OR = 0.12; 95%CI 0.05–0.31; *p* < 0.001 and OR = 0.38; 95%CI 0.15–0.98; *p* = 0.046, respectively); high stress (third tertile) was associated with medium and high self-efficacy (OR = 0.25; 95%CI 0.09–0.67; *p* = 0.006 and OR = 0.05; 95%CI 0.02–0.15; *p* < 0.001, respectively) and negative impact of COVID-19 in family finances (OR = 3.00; 95%CI 1.39–6.44; *p* = 0.005). It was concluded that parents/caregivers’ perceived stress was averaged and associated with self-efficacy, finger/nail biting, oral hygiene demands, and the financial impact of COVID-19.

## 1. Introduction

Down syndrome (DS) is a genetic disorder characterized by the presence of an extra chromosome in the pair 21. Among the most prevalent clinical and systemic alterations in DS are immune, cardiovascular, and intellectual impairment and reduced muscle tone [1,2]. Individuals living with DS may also have alterations in dental development, malocclusion, changes in the tongue, mouth breathing, and periodontal diseases, among others [3,4,5,6,7,8]. The oral health conditions of individuals with DS not only affect their quality of life but also that of their family members [5,9].

The family of individuals living with DS often demonstrates a high level of stress associated with both the diagnosis of the syndrome and with the difficulties experienced in everyday life, and also the great demand for care and treatment directed at these individuals. Health-related problems can affect their general well-being and their families [4,10], with an impact on stress levels, especially for parents/caregivers [11].

In addition to these systemic and dental challenges, in recent years, a greater concern for the population with DS has been present, considering the outcry of the COVID-19 pandemic context. Since it is a new disease, data on SARS-CoV-2 virus infection in individuals with DS were limited [12], and this pandemic has further increased the pressure on caregivers of people with disabilities [13], exacerbating stress and increasing care burdens and health concerns. The COVID-19 pandemic has had a profound impact on the mental and emotional well-being of caregivers, especially for those caring for individuals with disabilities like DS. The uncertainty surrounding the virus, compounded by limited data on how SARS-CoV-2 affects individuals with DS, has added to the psychological burden on families [14].

A family-centered approach should be considered; therefore, assessing stress and associated factors that impact the care burden on the psychological health of parents/caregivers is important. Understanding the health and well-being of these individuals also benefits those who need to be taken care of [13]. Understanding the relationship between stress and coping strategies can inform clinical practices and support plans for individuals with DS and their families [15].

Self-efficacy is a coping characteristic that consists of people’s belief or confidence in their ability to successfully perform the necessary behavior to produce desirable outcomes [16,17] and can be an important tool to optimize health outcomes and others, including oral hygiene practices [18]. Self-efficacy was associated with stress in caretaking among mothers of newborns [19] and premature babies [20]. Moreover, self-efficacy was also associated with stress in different nursing scenarios [21,22,23,24], including the COVID-19 pandemic [25,26]. In this sense, self-efficacy may be a key coping measure when planning strategies to reduce perceived stress [22,23,26]. Furthermore, a survey demonstrated that self-efficacy interacted with COVID-19-related experiences such that higher levels of self-efficacy mitigated the effects of COVID-19-related experiences on parenting stress [27].

To date, there are no known studies that have investigated the association between perceived stress and perceived general self-efficacy among parents/caregivers of people with Down syndrome, also considering the COVID-19 pandemic context. We hypothesize that higher levels of self-efficacy in parents/caregivers are associated with lower perceived stress, especially in the context of increased oral care demands and the disruptions caused by the COVID-19 pandemic. Since oral health is a critical concern for individuals with DS, understanding how caregiver stress impacts oral care can inform interventions that improve both caregiver well-being and care outcomes. Thus, this study aimed to evaluate the perceived stress of parents/caregivers of individuals living with DS and its association with dental and behavioral variables and general self-efficacy, taking the COVID-19 pandemic context into consideration during the daily activities of parents/caregivers of individuals living with DS and their families.

## 2. Materials and Methods

### 2.1. Study Design and Sampling Strategy

A cross-sectional study was developed with a convenience sample of parents/caregivers of individuals with DS ranging from newborns to 56 years of age, who attend a non-governmental institution that assists people with disabilities (Parents and Friends of individuals with DS Association—APAE) from 97 municipalities in the state of Minas Gerais, Brazil. A convenience sample was selected due to the focus on a specific population and logistical constraints, especially those imposed by the pandemic. The study was approved by the Research Ethics Committee of the Federal University of Minas Gerais (CAAE protocol #22814919.5.0000.5149) on 3 December 2019.

Data were collected between July and November 2021. Parents/caregivers answered a structured questionnaire in an electronic online form. During this period of time, efforts were made to reach a higher number of participants in the survey. Invitations to participate in the research were carried out by the Interdisciplinary Center for Studies and Research of the Federation of APAEs in the state of Minas Gerais through a letter sent to all units in the state. Each unit identified the families of individuals living with DS enrolled at the institution, and contacts were made by letters of invitation, telephone calls, and text messages. This invitation was reinforced monthly during the collection period in order to increase participation in the study and minimize selection bias. In addition, an understandable and inclusive language was used in the questionnaire to improve participation. Inclusion criteria included being a primary caregiver of an individual with DS and willingness to participate. Exclusion criteria involved caregivers who could not complete the questionnaire due to literacy barriers or lack of access to the Internet.

### 2.2. Data Collection Instruments

#### 2.2.1. Structured Questionnaire

The questionnaire was adapted from the instrument used by Oliveira et al. [4] to assess specific stress and self-efficacy variables relevant to the caregiving context, particularly in light of changes brought about by the COVID-19 pandemic. It was composed of questions about individuals living with DS and their parents/caregivers related to individual and sociodemographic characteristics, behavioral habits, and medical and dental history. Questions related to the impact of COVID-19 on daily activities were included to capture potential changes in family dynamics, caregiving responsibilities, and financial stability due to the pandemic. Then, the occurrence of changes in daily family life, hygiene, and eating habits; the oral health of the child with DS; and family income were evaluated. The answer options for these questions were: (a) yes, there were positive changes; (b) there were no changes; (c) yes, there were negative changes. It is important to state that the structured questionnaire [4] and its included questions were tested in the pilot study, but the survey was not validated.

#### 2.2.2. Perceived Stress Scale

Perceived stress by parents/caregivers was assessed using the short version of the Perceived Stress Scale (PSS-10) [28]. In PSS-10, 4 items have positive questions being scored reversely, and 6 items have negative questions having their scores added directly. The score can range from 0 to 40. The higher the total score, the higher the PSS-10. The instrument was validated for Brazilian Portuguese by Luft et al. [29].

#### 2.2.3. Perceived General Self-Efficacy Scale

The self-efficacy of parents/caregivers was assessed using the short version of the General Perceived Self-Efficacy Scale (GPSS), which has 10 items with four response options: (1) It is not true about me; (2) It is hardly true of me; (3) It is moderately true of me; and (4) It is completely true of me. The final total score on the scale can range from 10 to 40. The higher the value, the greater the overall perceived self-efficacy. The instrument was validated for Brazilian samples by Souza and Souza [30].

It is important to state that the PSS-10 and GPSS scales were selected due to their validated use in assessing stress and self-efficacy in a caregiving context, making them suitable for the study population.

### 2.3. Pilot Study

The pilot study was carried out between May and June of 2021 at APAE in the municipality of Pará de Minas, Minas Gerais, Brazil. Twenty parents/caregivers participated and answered the online questionnaire. The aim of this stage of the study was to assess the respondents’ understanding and the time taken to complete the questionnaire. After analyzing the pilot study, the main study began.

The pilot study results were analyzed for response time, clarity of questions, and participant feedback on the questionnaire. The time that respondents spent for answers was approximately 30 min. Minor modifications were made to improve clarity, but no critical changes were necessary. As the modifications in this step were not critical and without interference with the collected information, and the pilot sample was representative of the larger study population, the data of the pilot study were included in the analysis of the main study to increase statistical power.

### 2.4. Statistical Analysis

A descriptive analysis was initially performed to characterize the sample. According to the main variable of interest (perceived stress), the sample was divided into three equal groups: (1) 1st tertile: low stress = scores 0–16 (11.89 ± 3.75); (2) 2nd tertile: medium stress = scores 17–20 (18.67 ± 1.15); (3) 3rd tertile: high stress = scores 21–40 (24.18 ± 3.63).

Perceived general self-efficacy was also divided into tertiles and considered as low (1st tertile = scores 0–30; 26.65 ± 4.93; *n* = 71), medium (2nd tertile = scores 31–35; 33.03 ± 5.58; *n* = 85), and high (3rd tertile = scores 36–40; 38.48 ± 1.45; *n* = 84).

Tertiles were used to categorize stress and self-efficacy levels to facilitate comparison across varying degrees of stress perception and self-efficacy within the sample.

Groups were compared for individual, sociodemographic, and behavioral variables of interest using the Chi-square and Mann–Whitney tests when appropriate. The age of individuals with DS was dichotomized (≤18 vs. >18 years) for the analysis to capture differences in stress levels when caring for minors versus adults with DS, as caregiving demands may shift with age. Ethnicity was categorized using the criteria of the Brazilian Institute of Geography and Statistics (IBGE) [31] for skin color and grouped into “white” and “mixed” for analysis purposes. Variables associated with perceived stress were evaluated by a multinomial logistic regression, using low stress as the reference category (1st tertile). Two separate analyses were performed, excluding and including the variables on the impact of the COVID-19 pandemic. All variables were included in the respective models and manually removed step by step, observing the changes in the coefficients and considering the significance of *p* < 0.05. During stepwise multinomial logistic regression, variables with the least impact on model fit, as indicated by changes in coefficients and non-significant P-values, were systematically removed. Potential confounding variables, including socioeconomic status and access to healthcare, were included in the regression models to minimize bias.

Analyses were performed using the Statistical Package for Social Sciences software (SPSS for Windows, version 21.0, IBM Corp, Armonk, NY, USA). The results were considered significant if *p* < 0.05.

## 3. Results

Overall, this present study found that perceived stress among parents/caregivers was associated with multiple sociodemographic, dental, and behavioral variables, as well as the impact of the COVID-19 pandemic.

A total of 257 parents/caregivers [age 48.69 ± 13.26 years] of individuals living with DS [age 18.71 ± 14.012 (0–56)] participated in the study. Most participants claimed to be the mothers of individuals with DS (82.9%) and to live with a partner (59.84%). Among the parents/guardians, 71.65% of them were classified in the group with low family income (≤2 Brazilian minimum wages) and 42.97% in the group with low educational level (<8 years of study). Most individuals living with DS were male (56.05%) and white (60.23%).

The total score for perceived stress was 17.84 ± 5.75 (0–39; median 18), and the overall perceived self-efficacy was 33.03 ± 5.57 (0–40; median 33).

The association between perceived stress and sociodemographic variables and general perceived self-efficacy can be seen in Table 1. Perceived stress in parents/caregivers was associated with the age of individuals living with DS (higher scores for individuals with DS ≤ 18 years of age) (*p* = 0.027), the absence of a helper in housework (which refers to any additional support in managing daily caregiving tasks) (*p* = 0.016) and general perceived self-efficacy (*p* < 0.001). Higher averages of the self-efficacy score were observed among parents/caregivers in the group classified as low stress.

After analyzing sociodemographic variables, we then explored the association between perceived stress and dental habits of individuals with DS. The association between perceived stress and dental variables and habits can be seen in Table 2. Perceived stress was associated with the habit of finger/nail biting by the individuals with DS (*p* = 0.029), perceived halitosis (*p* = 0.035), difficulties in performing oral hygiene (related to behavior management and brushing techniques) (*p* = 0.027), and the one responsible for this activity (*p* = 0.033), as well as the negative perception of the oral health of the DS individual by parents/caregivers (*p* = 0.017).

The association between perceived stress and the impact of the COVID-19 pandemic on daily activities can be seen in Table 3. Perceived stress was associated with changes in daily living with these individuals (*p* = 0.033), changes in their oral hygiene habits (*p* = 0.036), and changes in family income (*p* = 0.024).

Table 4 shows the results of the multinomial logistic regression. The final models are as follows, without considering pandemic context variables: Model 1—average stress (2nd tertile) was associated with the habit of biting nails by individuals living with DS (OR = 2.15; *p* = 0.024), difficulty in oral hygiene (OR = 2.40; *p* = 0.010), and a protective effect of general perceived self-efficacy (mean self-efficacy OR = 0.12; *p* < 0.001; high self-efficacy OR = 0.39; *p* = 0.049). Model 2—high stress (3rd tertile) was associated with difficulty in oral hygiene (OR = 2.21; *p* = 0.031) and a protective effect of general perceived self-efficacy (mean self-efficacy OR = 0.25; *p* = 0.005; high self-efficacy OR = 0.06; *p* < 0.001). In the final models, considering the context of the pandemic, mean stress (2nd tertile) remained associated with the same variables with small changes in OR estimates (model 3). However, high stress (3rd tertile) (model 4) was associated with a protective effect of overall perceived self-efficacy (mean self-efficacy OR = 0.25; *p* = 0.006; high self-efficacy OR = 0.05; *p* < 0.001) and the negative impact of the pandemic on family finances (OR = 3.00; *p* = 0.005).

Thus, it was observed that the COVID-19 pandemic significantly increased stress levels in parents/caregivers, particularly due to financial strain. Caregivers experiencing negative changes in family income were three times more likely to report high stress. Moreover, an OR of less than 1 for self-efficacy indicates a protective effect, meaning that higher self-efficacy is associated with reduced odds of experiencing high stress. It is important to highlight that, in all models, self-efficacy demonstrated a strong protective effect, with high self-efficacy consistently associated with lower stress levels (model 1: OR = 0.39; *p* = 0.049; model 2: OR = 0.06; *p* < 0.001; model 3: OR = 0.38; *p* = 0.046; model 4: OR = 0.05; *p* < 0.001). The logistic models controlled for potential confounders, including socioeconomic status and education level, to isolate the effects of self-efficacy and caregiving demands on perceived stress. The median values of stress and self-efficacy provide a reference point for the distribution of scores, helping to classify caregivers into low, medium, and high-stress groups.

## 4. Discussion

Findings from the present study indicated that the perceived stress among parents/caregivers was associated with difficulties in performing oral hygiene of individuals with DS, that the COVID-19 pandemic significantly increased stress levels particularly due to financial strain, and that self-efficacy had a strong protective effect, with high self-efficacy consistently associated with lower stress levels.

As highlighted in this study, caregiving for individuals with Down syndrome (DS) can be highly demanding, especially when family support is limited, contributing to increased levels of perceived stress. The daily routine of a family of people with disabilities can have a high demand for special care, including the ones living with DS. Often, if there is no family support, the main caregiver may have an overload of activities that result in high levels of stress [12,32,33].

In this present study, higher perceived stress scores were associated with younger ages of individuals living with DS. Previous studies highlight the importance of age in relation to the individual’s daily needs and the stress and burden of families of individuals with DS [33,34]. Older ages and better self-care skills of individuals with DS were protective factors for the mental health of their parents/caregivers. Strategies focused on improving self-care skills can favor the development of people with disabilities and reduce depression, stress, and tension in the ones responsible for these individuals [14]. While previous studies have established the link between caregiving stress and the age of individuals with DS, this study adds to the literature by highlighting the protective role of autonomy in reducing caregiver stress, particularly regarding oral hygiene practices.

The perceived stress of parents/caregivers was associated with a negative perception of oral health, perceived halitosis, and demands of oral hygiene practices, such as difficulties in toothbrushing and who performs it. It was previously reported that more than 60% of parents/caregivers find it difficult to brush the teeth of these care-given individuals [35]. The main reasons why they dislike oral hygiene are the feeling of the toothbrush in the mouth, the taste or texture of the toothpaste, the need for physical restraint, and gingival bleeding during the procedure performed by those who were held responsible for them [35].

Although oral health care can be laborious and stressful for parents/caregivers, it should be encouraged as it is an important measure for controlling dental biofilm, the etiological agent that determines oral diseases such as caries and periodontal disease. The results presented here indicate that when individuals with DS are older and carry out their own oral hygiene care, the perceived stress of their parents/caregivers is lower. Therefore, autonomy for this group can greatly benefit the entire family nucleus. Given the challenges associated with oral hygiene in individuals with DS, targeted interventions aimed at improving their self-care skills could significantly reduce the stress burden on caregivers.

It is necessary that oral health care professionals are aware of the importance of promoting favorable behavior to the patient’s oral health and psychological theories, such as the theory of self-efficacy. A study with normotypical adolescents showed that oral self-care skills were superior when guided by the self-efficacy theory compared to conventional methods [18]. In the self-efficacy theory [16], outcome expectations stem from our expectations of actions and results, which can be optimized when we are motivated and trained to develop skills, such as hygiene practices [18]. Assertive strategies should include adequate counseling and guidance to parents/caregivers and family members [5,9].

Higher perceived stress scores were associated with lower perceived general self-efficacy scores. These findings are in line with other findings in the literature that indicate a negative correlation between self-efficacy and perceived stress [22] and anxiety [36]. Furthermore, a direct correlation between self-efficacy and coping and problem-solving strategies has been reported [36]. Therefore, it is possible that the self-efficacy of parents/caregivers can also reduce their burden and stress. The protective effect of it was reinforced in multivariate models where higher perceived stress scores were associated with lower self-efficacy scores, considering the context of the COVID-19 pandemic. The findings that higher self-efficacy is strongly associated with lower stress suggest that interventions aimed at increasing caregiver self-efficacy, such as skills training and psychological support, could be valuable in reducing caregiver burden.

In this study, perceived stress was associated with the finger/nail biting habit of individuals with DS, a habit that is associated with stress [37]. Although we have not evaluated the stress of people with DS, it is assumed that its presence may reflect the anxiety of these individuals and have repercussions for their core family members. The presence of this deleterious habit has previously been associated with the development of malocclusion in children and adolescents with DS [38]. Malocclusion is an oral health problem associated with a negative impact on the oral health-related quality of life of family members of this group of patients [9]. Though we did not carry out clinical examination and assessment of the presence of malocclusion, we can believe that this is a factor capable of altering the perception of oral health of individuals with DS by parents/caregivers based on the available evidence.

Although perceived stress was not directly associated with sociodemographic factors such as family income or level of education of parents/caregivers, the negative impact of the COVID-19 pandemic on family finances and the absence of a helper to carry out household chores were associated variables with perceived stress that can indirectly express family financial conditions. It was also previously reported that there was no association between the stress of mothers of people with DS and sociodemographic factors, but unemployment was a factor that increased the level of stress [15]. The financial strains caused by the COVID-19 pandemic were significantly associated with higher stress levels. Policymakers and support organizations should prioritize financial assistance and access to support services for families affected by this economic disruption.

It is important to consider that, during the pandemic, lifestyles and daily routine paces were interrupted by the loss of support for families due to social isolation. Individuals living with DS had to be absent from school and often stopped seeing family and friends who were part of their lives [39]. Caregivers have previously reported important impacts of some demographic and behavioral factors upon oral care/habits during the COVID-19 pandemic, although children’s oral health status did not suffer noteworthy alterations [40]. In this present study, a higher percentage of caregivers reported a negative impact on the oral hygiene habits of individuals with DS in the high-stress group when compared to the other groups. Indeed, family functioning, resilience, and stress were positively correlated with general hygiene and oral hygiene habits [41]. Health systems needed to focus on essential care, and under these circumstances, specialized treatment centers had to be temporarily closed, leaving this portion of the population without specialized monitoring and specific guidance [42], including dental care.

Some studies have already demonstrated the importance of health care for parents/caregivers of children with disabilities [14], with cerebral palsy [13], and with developmental disabilities such as autism spectrum disorder and attention deficit hyperactivity disorder [43], especially in the context of the COVID-19 pandemic. However, to date, we are unaware of a study that associated perceived stress by parents/caregivers of individuals living with DS and the COVID-19 pandemic. Its influence on the lives of children and their caregivers is likely to continue well beyond its resolution, and follow-up studies may reveal a range of long-term consequences [43].

The hypothesis that the level of perceived stress of parents/caregivers may be associated with self-efficacy (so that the higher the level of perceived general self-efficacy, the lower their perceived stress index) and that difficulties in oral hygiene and oral health care can be factors associated with stress were confirmed.

The perceived stress level of parents/caregivers of individuals living with DS in this present study was average, but these results should be interpreted with caution and should not be extrapolated to parents/caregivers of individuals with other disabilities, as these estimates may be different [44]. Specific sample characteristics should be considered since the sociodemographic profile of families assisted by institutions for people with disabilities may be different from families living with this population with DS, who have their children enrolled in regular schools and are a reality today due to advances in social inclusion. In addition, some limitations of the study should be pointed out, as it is a cross-sectional design with a convenience sample. The use of a cross-sectional design limits the ability to establish causal relationships, and the convenience sample may not fully represent the broader population of caregivers for individuals with DS. Future research using longitudinal designs and more diverse samples would provide a more comprehensive understanding of the associations reported in this study. It is also important to point out that changes imposed by the COVID-19 pandemic and lockdown periods may have impacted family functioning and behaviors in different aspects, hence influencing the collected data to some degree. Nonetheless, this study points to an important direction of the association between perceived stress and general perceived self-efficacy.

## 5. Conclusions

The study found that the perceived stress levels of parents and caregivers of individuals with Down syndrome were moderate and significantly associated with lower self-efficacy scores and challenges in managing oral hygiene practices for individuals with DS. In the context of the COVID-19 pandemic, financial strain was an additional factor contributing to higher perceived stress among caregivers, highlighting the compounded challenges faced during this period. The results underscore the importance of developing coping strategies, particularly enhancing self-efficacy, to reduce the burden on parents and caregivers of individuals with DS. Interventions that build self-efficacy and provide support for managing daily caregiving tasks, including oral hygiene, should be prioritized and encouraged.

## Figures and Tables

**Table 1:** Association between sociodemographic and behavioral variables and perceived stress by parents/caregivers of individuals with DS.

Variables	Perceived Stress			*p*
	Low (1° Tertile)	Medium (2° Tertile)	High (3° Tertile)	
Sex of the individual with DS				
Male	51 (56.7%)	55 (58.5%)	37 (52.1%)	0.766
Female	39 (43.3%)	39 (41.5%)	34 (47.9%)	
Age of the individual with DS (years)	22.5 ± 15.2	16.8 ± 12.6	16.6 ± 13.5	0.015
Age of the individual with DS				
≤18 years	35 (39.8%)	55 (57.9%)	41 (56.9%)	0.027
>18 years	53 (60.2%)	40 (42.1%)	31 (43.1%)	
Ethnicity of the individual with DS				
White	51 (56.7%)	53 (57.6%)	49 (68.1%)	0.275
Mixed	39 (43.3%)	39 (42.4%)	23 (31.9%)	
Family income				
≤2 Brazilian minimum wages *	58 (64.4%)	72 (76.6%)	52 (74.3%)	0.159
≥3 Brazilian minimum wages *	32 (35.6%)	22 (23.4%)	18 (25.7%)	
Parents/caregiver’s marital status				
With companion	40 (44.4%)	31 (33.0%)	31 (44.3%)	0.202
Without companion	50 (55.6%)	63 (67.0%)	39 (55.7%)	
Educational level of parents/caregivers				
<8 years	39 (43.3%)	38 (40.4%)	33 (45.8%)	0.962
8 to 11 years	28 (31.1%)	32 (34.0%)	21 (29.2%)	
≥12 years	23 (25.6%)	24 (25.5%)	18 (25.0%)	
Occupation of parents/caregivers				
Household chores solely	48 (53.9%)	58 (61.1%)	51 (70.8%)	0.091
Household and professional chores	41 (46.1%)	37 (38.9%)	21 (29.2%)	
Presence of a helper in housework				
No	79 (88.8%)	94 (98.9%)	67 (93.1%)	0.016
Yes	10 (11.2%)	1 (1.1%)	5 (6.9%)	
Self-Efficacy Score	35.8 ± 4.6	32.4 ± 4.8	30.5 ± 6.2	<0.001
Self-Efficacy				
Low	8/9 (9.6%)	31/33 (34.8%)	32/34 (47.1%)	<0.001
Medium	25/27 (30.1%)	36/38 (40.4%)	24/26 (35.3%)	
High	50/53 (60.2%)	22/24 (24.7%)	12/13 (17.6%)	

* Brazilian minimum wage equivalent to USD 250.

**Table 2:** Association between dental variables and habits and perceived stress by parents/caregivers of individuals with DS.

Variables	Perceived Stress			*p*
	Low (1° Tertile)	Medium (2° Tertile)	High (3° Tertile)	
Habit of sticking the tongue out				
No	57 (63.3%)	48 (50.5%)	35 (48.6%)	0.108
Yes	33 (36.7%)	47 (49.5%)	37 (51.4%)	
Drooling habit				
No	74 (82.2%)	73 (76.8%)	52 (72.2%)	0.314
Yes	16 (17.8%)	22 (23.2%)	20 (27.8%)	
Habit of biting finger/nails				
No	54 (60.7%)	39 (41.1%)	37 (51.4%)	0.029
Yes	35 (39.3%)	56 (58.9%)	35 (48.6%)	
Gingival bleeding				
No	62 (69.7%)	57 (62.6%)	43 (64.2%)	0.587
Yes	27 (30.3%)	34 (37.4%)	24 (35.8%)	
Toothache				
Never	64 (71.9%)	58 (69.9%)	45 (73.8%)	0.846
Sometimes	23 (25.8%)	21 (25.3%)	13 (21.3%)	
Frequently	2 (2.2%)	4 (4.8%)	3 (4.9%)	
Perceived halitosis				
No	59 (65.6%)	53 (76.8%)	42 (58.3%)	0.035
Yes	31 (34.4%)	22 (23.2%)	30 (41.7%)	
Toothbrushing frequency				
1× a day	8 (8.9%)	12 (12.9%)	12 (16.9%)	0.140
2× a day	33 (36.7%)	47 (50.5%)	31 (43.7%)	
3× a day or more	49 (54.4%)	34 (36.6%)	28 (39.4%)	
Flossing				
No	51 (56.7%)	43 (46.2%)	29 (40.8%)	0.119
Yes	39 (43.3%)	50 (53.8%)	42 (59.2%)	
Tongue brushing				
No	23 (27.4%)	31 (35.6%)	22 (31.4%)	0.510
Yes	61 (72.6%)	56 (64.4%)	48 (68.6%)	
Difficulties in performing oral hygiene				
No	54 (60.0%)	40 (42.6%)	30 (42.3%)	0.027
Yes	36 (40.0%)	54 (57.4%)	41 (57.7%)	
Who performs oral hygiene?				
Parents/caregivers	24 (26.7%)	48 (50.0%)	32 (44.4%)	0.033
Individuals with DS that were supervised by parents/caregivers	22 (24.4%)	17 (17.9%)	18 (25.0%)	
Individuals with DS	44 (48.9%)	30 (31.6%)	22 (30.6%)	
Perception of oral health by the parents/caregivers				
Positive	66 (74.2%)	55 (58.5%)	37 (53.6%)	0.017
Negative	23 (25.8%)	39 (41.5%)	32 (46.4%)	

**Table 3:** Association between the impact of the COVID-19 pandemic and perceived stress by parents/caregivers of individuals with DS.

Variables	Perceived Stress			*p*
	Low (1° Tertile)	Medium (2° Tertile)	High (3° Tertile)	
The impact of the pandemic on daily contact with individuals living with DS				
For better	24 (26.7%)	30 (31.9%)	22 (30.6%)	0.033
For worse	9 (10.0%)	7 (7.5%)	17 (23.6%)	
No changes	57 (63.3%)	57 (60.6%)	33 (45.8%)	
The impact of the pandemic on the oral hygiene habits of individuals living with DS				
For better	18 (20.0%)	18 (18.9%)	18 (25.4%)	0.036
For worse	3 (3.3%)	4 (4.2%)	10 (14.1%)	
No changes	69 (76.7%)	73 (76.8%)	43 (60.6%)	
The impact of the pandemic on the oral health of individuals with DS				
For better	3 (3.3%)	6 (6.4%)	4 (5.2%)	0.088
For worse	9 (10.0%)	6 (6.4%)	15 (21.1%)	
No changes	78 (86.7%)	82 (87.2%)	52 (73.2%)	
The impact of the pandemic on the eating habits of individuals with DS				
For better	11 (12.2%)	16 (17.2%)	9 (12.7%)	0.591
For worse	17 (18.9%)	18 (19.4%)	19 (26.8%)	
No changes	62 (68.9%)	59 (63.4%)	43 (60.5%)	
Impact of the pandemic on family finances				
For better	1 (1.1%)	2 (2.1%)	2 (2.8%)	0.024
For worse	37 (41.1%)	45 (47.4%)	47 (65.3%)	
No changes	52 (57.8%)	48 (50.5%)	23 (31.9%)	

**Table 4:** Multinomial logistic regression models for perceived stress (reference category = low stress—1st tertile).

Variables	Coefficient	OR (95% IC)	*p*
Model 1: Medium stress (2° tertile)			
Habit of finger/nail biting	0.763	2.15 (1.11–4.18)	0.024
Difficulties in oral hygiene	0.875	2.40 (1.24–4.65)	0.01
Self-efficacy			
Low	reference	–	–
Medium	−2.091	0.12 (0.05–0.32)	<0.001
High	−0.954	0.39 (0.15–0.99)	0.049
Constant	0.507	–	0.28
Model 2: High stress (3° tertile)			
Habit of finger/nail biting	0.321	1.38 (0.67–2.85)	0.386
Difficulties in oral hygiene	0.796	2.21 (1.08–4.57)	0.031
Self-efficacy			
Low	reference		
Medium	−1.389	0.25 (0.09–0.66)	0.005
High	−2.834	0.06 (0.02–0.16)	<0.001
Constant	0.832	–	0.079
Model 3: Medium stress (2° tertile)			
Habit of finger/nail biting	0.717	2.05 (1.04–4.03)	0.038
Difficulties in oral hygiene	0.87	2.39 (1.23–4.65)	0.011
Self-efficacy			
Low	reference	–	–
Medium	−2.122	0.12 (0.05–0.31)	<0.001
High	−0.969	0.38 (0.15–0.98)	0.046
Impact of the pandemic on family			
finances			
No changes	reference	–	–
For worse	0.246	1.28 (0.65–2.34)	0.481
For better	0.37	1.45 (0.08–26.48)	0.803
Constant	−0.436	–	0.364
Model 4: High stress (3° tertile)			
Habit of finer/nail biting	0.139	1.15 (0.54–2.43)	0.715
Difficulties in oral hygiene	0.693	2.00 (0.96–4.18)	0.066
Self-efficacy			
Low	reference		
Medium	−1.390	0.25 (0.09–0.67)	0.006
High	−2.920	0.05 (0.02–0.15)	<0.001
Impact of the pandemic on family			
finances			
No changes	reference	–	–
For worse	1.096	3.00 (1.39–6.44)	0.005
For better	1.08	2.94 (0.15–56.49)	0.474
Constant	0.401	–	0.426

Models 1 and 2: without considering the context of the pandemic; models 3 and 4: considering the context of the pandemic.

## Data Availability

Datasets supporting the study results are available from the corresponding author upon reasonable request.
